# Occurrence of *Toxoplasma gondii* and *Neospora caninum* antibodies and risk factors in domiciliated dogs of Manaus, Amazonas, Brazil

**DOI:** 10.1590/S1984-29612022024

**Published:** 2022-05-09

**Authors:** Hevila Gabrieli Nascimento de Campos, Herbert Sousa Soares, Sergio Santos de Azevedo, Solange Maria Gennari

**Affiliations:** 1 Programa de Pós-graduação em Saúde Única, Faculdade de Medicina Veterinária, Universidade Santo Amaro – UNISA, São Paulo, SP, Brasil; 2 Unidade Acadêmica de Medicina Veterinária, Universidade Federal de Campina Grande – UFCG, Patos, PB, Brasil

**Keywords:** Toxoplasma gondii, Neospora caninum, antibodies, risk factors, dogs, Toxoplasma gondii, Neospora caninum, anticorpos, fatores de risco, cães

## Abstract

The presence of antibodies anti-*Toxoplasma gondii* and *Neospora caninum* have been described in dogs from virtually all Brazilian states, however in the state of Amazonas, there are few studies on these coccidia. In this study the occurrence of antibodies against *T. gondii* and *N. caninum* and risk factors were determined in domiciliated dogs of Manaus, AM. Blood samples were collected from 154 dogs and, during the harvest, a questionnaire was applied with questions related to the animals. The samples were analyzed, for the presence of anti-*T. gondii* and *N. caninum* antibodies, by indirect fluorescence antibody test, with cutoff of 16 and 50, respectively. Associations between the variables studied and the presence of antibodies were made by chi-square test, fisher's exact test or G test (p<0.05). Of the 154 samples, 19 (12.3% 95% CI = 7.1% - 17.5%) were reagents to *T. gondii,* and association (p <0.05) was observed between the presence of antibodies and contact with other dogs. The occurrence of dogs reactive to *N. caninum* was 1.9% (95% CI = 0.4% - 5.6%) with 3 of the 154 dogs positives, and no association (p>0.05) was observed between the presence of *N. caninum* antibodies, and the variables studied.

## Resumo

A ocorrência de anticorpos anti-*Toxoplasma gondii* e anti-*Neospora caninum* já foram descritos em cães de praticamente todos os estados brasileiros, entretanto, no estado do Amazonas, há poucos estudos sobre esses coccídios. Neste estudo, a ocorrência de cães domiciliados de Manaus, reagentes a *T. gondii* e *N. caninum*, e os fatores de risco para a infecção foram avaliados. Amostras de sangue de 154 cães foram obtidas, e um questionário foi aplicado aos tutores com informações sobre os animais. As amostras foram analisadas quanto à presença de anticorpos anti-*T. gondii* e *N. caninum*, pela reação de imunofluorescência indireta, ponto de corte 16 e 50, respectivamente. Associações entre as variáveis estudadas e a presença de anticorpos contra os coccídio*s* foram feitas pelo teste de qui-quadrado, exato de Fisher ou G (p<0,05). Das 154 amostras, 19 (12,3%; IC 95% = 7,1% - 17,5%) foram reagentes a *T. gondii*, e a associação (p <0,05) foi observada entre a presença de anticorpos e contato com outros cães. A ocorrência de cães reagentes a *N. caninum* foi de 1,9% (IC 95% = 0,4% - 5,6%) com 3 dos 154 cães positivos. Nenhuma associação (p>0,05) foi observada entre a presença de anticorpos anti-*N. caninum* e as variáveis estudadas.

Toxoplasmosis is an important zoonosis, caused by the protozoan of the Apicomplexa phylum, *Toxoplasma gondii*, common worldwide and highly prevalent in humans and animals in Brazil (Dubey et al., 2012). Felids are the only definitive hosts of this coccidia, where the sexual cycle occurs, and which excrete oocysts through feces and more than 350 species of mammals and birds have already been described as intermediate hosts (Dubey et al., 2012). The hosts, including humans, can become infected by ingesting food or water contaminated with oocysts or by consuming tissue cysts of *T. gondii*, present in raw or undercooked meat, from animals infected with coccidia. There is also an important form of infection, the transplacental, which occurs when the mother becomes infected during pregnancy and the forms of rapid multiplication of the parasite, the tachyzoites, reach the fetus (Dubey et al., 2012).

Dogs reactive to *T. gondii* have already been described in practically all Brazilian states (reviewed by Dubey et al., 2012, 2020) and, in the state of Amazonas (AM), there is a study in the city of Manaus, from 1980, in which the occurrence of antibodies against *T. gondii* was evaluated in dogs by the hemagglutination test, with a value of 68%, with 13 of the 19 dogs examined being reactive (Ferraroni & Marzochi, 1980). In a more recent study, carried out in the city of Lábrea, AM, 99 dogs were examined for the presence of anti-*T. gondii* antibodies and 61.6% were reactive (Basano et al., 2016), confirming the occurrence of the parasite in dogs in the region.

A review on toxoplasmosis in Brazil with studies from 1968 to 2012 (Dubey et al., 2012), as well as a more recent review on *T. gondii* in dogs in the world (Dubey et al., 2020) presents data on the occurrence of antibodies in domiciled and stray dogs, and in the Brazilian domiciled dogs the values ranged from 3.1% to 91%, however, due to the different methodologies and cut-off used, comparisons should be made with care.

The coccidia Apicomplexa *Neospora caninum*, which causes neosporosis, is considered, in some regions of the world, as the main cause of abortions in cattle. In dogs, especially in neonates, it can also cause miscarriages and severe neuromuscular disease (Dubey et al., 2007). Morphologically it is a coccidia very similar to *T. gondii*, but different biologically. It is not considered a zoonotic agent, although antibodies against *N. caninum* have already been found in humans, the parasite was not detected in tissues (Lobato et al., 2006; Oshiro et al., 2015). More recently IgG antibodies to *N. caninum* were detected, by immunofluorescence assay and PCR, in human umbilical cord blood and a significant association were observed between *N. caninum* seropositivy and the presence of domestic animals and presence of dogs, however none of the sampled placenta showed structures characteristic of tissue cysts or infammatory infltrate on histopathology (Duarte et al, 2020).

*N. caninum* has as definitive host the dog (McAllister et al., 1998) and some genera of wild canids, such as coyotes (Gondim et al., 2004) and gray wolves (Dubey et al., 2011), and several warm-blooded animals as intermediate hosts. Canids eliminate non-sporulated oocysts in their feces that, via food and water, can infect several other animal species or it can be transmitted transplacentally (vertically, congenitally), from an infected dam to her foetus during pregnancy (Cerqueira-Cézar et al., 2017).

In a study carried out in the Amazon region, with samples from the Brazilians states of Mato Grosso and Tocantins, with dogs from indigenous villages, the occurrence of anti-*N. caninum* antibodies was 9.8%, with 32 of the 325 dogs examined positive (Minervino et al., 2012). In the state of Pará, also in the Brazilian Amazon region, urban and rural dogs were examined for antibodies against *N. caninum*, with 14% and 11.1% of positivity, respectively, and no significant difference was observed between dogs that lived in the different areas (Valadas et al., 2010).

The aim of the present study was to determine the occurrence of antibodies against *T. gondii* and *N. caninum* in domiciliated dogs in the city of Manaus, AM, as well as the risk factors associated with these infections.

The study was conducted at University Santo Amaro, São Paulo, SP, Brazil, with approval of the Committee on Ethics in the Use of Animals (CEUA) number 20/2020. The owners of the dogs who participated in the study signed an informed consent statement that described the procedures to be on the dogs.

The city of Manaus, capital of the state of Amazonas, Brazil covers an area of ​​about 11,401 km^2^, an estimated 2,219,580 inhabitants, and its geographic coordinates are 03°06'07”S and 60°01'30”W (IBGE, 2021). The collections of the samples were carried out by convenience sampling, in the homes, to cover the entire city, which is divided into six zones: North (ZN), South (ZS), Central South (ZCS), East (ZL), West (ZW) and Midwest (ZMW).

The minimum sample size was calculated based on the simple random sample formula, with an expected prevalence of 50% for sample maximization, confidence level of 95%, and sampling error of 10% (Thrusfield & Christley, 2018). The formula showed that the minimum number of animals was 96. However, blood samples were collected, by convenience, from 154 domiciliated dogs, male and female, of different breeds, young (0-12 months) and adults (>12 months).

Although the samples were obtained for convenience, care was taken to obtain samples from domiciled dogs, from all areas of the municipality and from animals of different ages. The samples were collected from the jugular or cephalic vein, the blood was placed in a tube with separating gel (BD SST™ II Vacutainer®) and subsequently centrifuged at 700 x g for 10min. The serum was stored in Eppendorf® tubes, at -17°C. The collection period was from January to March 2021.

During the collection of the samples, a questionnaire was applied to guardians with questions related to age, gender, breed, contact with other dogs and cats, type of feeding, access to the streets, and region of dogs’ home.

From the total of 154 dogs, 59 (38.3%) were males and 95 (61.6%) were females; 99 (64.2%) were mixed-breed dogs and 55 (35.7%) purebred dogs; 131 (85.0%) remain exclusively in domestic environment and 23 (15%) were allowed by their owners to stay free outside the home. In relation to age, 25 (16.2%) were less than one years and 129 (83.8%) were more than one year old. Of the owners, 87 (56.5%) reported the dogs have contact with cats and 67 (43.5%) had no contact with cats. Sampled dogs with had contact with other dogs was 87 (56.5%), and 67 (43.5%) had no contact with other dogs. Animals were sampled in all the six zones of Manaus, been: 40 (25.9%) from North, 13 (8.4%) from South, 31 (20.1%) from East, 29 (18.8%) from West, 21 (13.6%) from Midwest and 20 (13%) from South-central (Table 1).

**Table 1:** Occurrence of antibodies for *T. gondii* and *N. caninum* in serum samples from domiciled dogs in the city of Manaus, Amazonas, Brazil, determined by means of the indirect fluorescence antibody test by analyzed variables.

**Variables**			**No. of dogs (%)**	**No. of positives (%)**				
				***T. gondii***			***N. caninum***	
**Sex**								
	Male		59 (38.3)	9 (15.2)			2 (3.4)	
	Female		95 (61.6)	10 (10.5)			1 (1.0)	
**Age**								
	0-12 months		25 (16.2)	3 (12.0)			0 (0.0)	
	>12 months		129 (83.8)	16 (12.4)			3 (2.3)	
**Breed**								
	Defined		55 (35.7)	4 (7.3)			0 (0.0)	
	Non defined		99 (64.2)	15 (15.1)			3 (20.0)	
**Food**								
	Commercial		131 (85.0)	14 (10.7)			3 (21.4)	
	Mixed		23 (15.0)	5 (21.7)			0 (0.0)	
**Contact with streets**								
	Yes		23 (15.0)	4 (17.4)			0 (0.0)	
	No		131 (85.0)	15 (11.5)			3 (20.0)	
**Contact with other dogs**								
	Yes		87 (56.5)	18 (20.7)*			3 (9.1)	
	No		67 (43.5)	**1 (1.5)**			0 (0.0)	
**Contact with cats**								
	Yes		87 (56.5)	11 (12.6)			0 (0.0)	
	No		67 (43.5)	8 (11.9)			3 (4.5)	
**Residence (zone)**								
	North		40 (25.9)	4 (10.0)			0 (0.0)	
	South		13 (8.4)	0 (0.0)			0 (0.0)	
	East		31 (20.1)	8 (25.8)			1 (3.2)	
	West		29 (18.8)	6 (20.7)			1 (3.4)	
	Central-West		21 (13.6)	2 (9.5)			0 (0.0)	
	Central-South		20 (13.0)	1 (5.0)			1 (5.0)	
**TOTAL**			**154 (100)**	**19 (12.3)**			**3 (1.9)**	

* Significant difference (p < 0.05) for *T. gondii*.

Sera were analyzed by Indirect Fluorescence Antibody Test (IFAT) for detection of antibodies against *T. gondii* and *N. caninum* according to Camargo (1974). Anti-dog IgG conjugate (Sigma®, USA), obtained from rabbits and labeled with fluorescein isothiocyanate, were diluted in 1:600, in phosphate buffer solution (PBS) pH 7.2 containing 0.01% Evans blue, was used.

For *T. gondii* tachyzoites of the RH strain were used as antigens and the cutoff value was 16 (Valadas et al., 2010). For *N. caninum* NC-1 strain tachyzoites, maintained in cell culture, were used as antigen and the cutoff point was 50 (Valadas et al., 2010). On each slide, control sera, positive and negative, were added. Reagent serum samples were titrated on base two until the last positive dilution was obtained.

Association between the studied variables (zone of residence, sex, age, breed, type of food, going to the streets, contact with other dogs and contact with cats) and the presence of anti-*T. gondii* and anti-*N. caninum* antibodies was performed using the chi-square test, Fisher's exact test or G test, with a significance level of 5%.

The occurrence ​​of *T. gondii* and *N. caninum* serum antibodies obtained for each variable analyzed are shown in Table 1. Of the 154 dogs examined, 19 (12.3%; 95% CI = 7.1%-17.5%) were reactive to *T. gondii* with titers of: 16 (n=5), 32 (n=9), 64 (n=1), 128 (n=2), 256 (n=1) and 512 (n=1) and only 3 (1.9%; 95% CI = 0.4% - 5.6%) dogs were reactive to *N. caninum* with titers of 800 (n=1), 200 (n=1) and 100 (n=1).

Among the variables analyzed, association (p <0.05) was observed between the presence of antibodies to *T. gondii* and contact with other dogs, with higher occurrence (20.7%) in those dogs when compared with dogs with no contact (1.5%). No association was found between the presence of dogs reactive to *N. caninum* and the studied variable (p >0.05).

The results indicate that dogs in the municipality of Manaus are exposed to *T. gondii* and *N. caninum* infection.

Seropositivity for *T. gondii* presented an association with dogs that living with other dogs or that have contact with dogs, that presented an occurrence more than ten times higher than that found in dogs that do not have this contact. Benitez et al. (2017), in a study with dogs and their owners in Londrina, PR, Brazil, observed that among dogs, the absence of other dogs and the absence of a dirty yard were concomitant significantly protective associated factors for *T. gondii* infection.

Although the *T. gondii* occurrence values ​​are much higher for dogs that feed on commercial food associated with homemade food when compared to feeding exclusively homemade food, this difference was not significant (p > 0.05; ꭓ^2^). Dogs that frequent the streets also presented a higher occurrence than dogs that do not have contact with the streets, however this difference was not statistically significant either (p > 0.05; ꭓ^2^).

Except for South area, one of the most developed in the city, in all other areas reactive dogs were found. According to de Oliveira & Costa (2007), the north, west and east zone of Manaus presented the lowest level of human development and in those zone high occurrence values of reagent dogs to *T. gondii* were observed. It is noteworthy that the flood and drought system, which constantly occurs in the Amazon River and in the Igarapes (Amazon tributaries) that crossing the entire city, and that frequently flood many regions of Manaus, probably dilutes the environmental contamination by oocysts and therefore, the animals’ exposure. However, the real effect of these changes between drought and flood on these parasitic forms, is not known and deserves future studies.

The *T. gondii* occurrence, found among the dogs in the present study, was lower (12.3%) than the rate of 68% found previously by Ferraroni & Marzochi (1980) in the same city, however very few dogs were examined in their study, and they used a different diagnostic test. In the Amazon region, studies of occurrence of *T. gondii* antibodies also presented higher rates than the results of the present study, with 61.6% in Lábrea, a city in the south of Amazon state (Basano et al., 2016), 76.5% in Rondônia (Cañón-Franco et al., 2004), and 69.8% and 38% in Pará (Valadas et al., 2010, Paz et al., 2019). However, despite all studies had been made in Amazon region and with the same diagnostic technique, the environmental and the characteristics of the dogs’ sampled are not the same.

This is the first description of dogs seropositive to *N. caninum* in the state of Amazon. The occurrence of 1.9% (95% CI = 0.4% - 5.6%) for neosporosis found in dogs in this study is lower than that reported for dogs from the other Amazon region, states of Mato Grosso and Tocantins, with 9.8% prevalence (Minervino et al., 2012). Also in the same region, in the state of Pará, urban stray dogs presented an occurrence of 14% to antibody to *N. caninum* (Valadas et al., 2010) and in Rondônia domiciliated dogs, that had street access, presented a prevalence of 8.3% (Cañon-Franco et al., 2003).

Studies in dogs from different regions of Brazil found that the age of the animals, contact with other dogs and feed habits are the more common risk factor for *N. caninum* infection (reviewed by Cerqueira-Cézar et al., 2017), however in the present study no studied variables presented association with dogs reactive to *N. caninum* antibodies.

Dogs are considered sentinels for *T. gondii* environmental contamination. Data on human toxoplasmosis in Manaus are very scarce and old. Ferraroni et al. (1980) described values of 70.6% in humans, however the samples were from only one region of the city and very few information is available about the sampled persons and location. Based on the observations found in this study it will be important a new epidemiological survey of this zoonosis in the municipality with human being, and other animal species, including stray dogs.

In relation to *N. caninum*, as a proportion of dogs showed evidence of exposure to the parasite, neosporosis should be considered in the differential diagnosis of neurological disorders by the veterinarians of the municipality, as well as non-neurological signs, such as myositis, dermatitis, pancreatitis, pneumonia, and hepatitis (Dubey et al., 2017).
